# Porphyrins produce uniquely ephemeral animal colouration: a possible signal of virginity

**DOI:** 10.1038/srep39210

**Published:** 2016-12-15

**Authors:** Ismael Galván, Pablo R. Camarero, Rafael Mateo, Juan J. Negro

**Affiliations:** 1 Departamento de Ecología Evolutiva, Estación Biológica de Doñana - CSIC, 41092 Sevilla, Spain; 2 Instituto de Investigación en Recursos Cinegéticos - CSIC-UCLM-JCCM, 13005 Ciudad Real, Spain

## Abstract

Colours that underlie animal pigmentation can either be permanent or renewable in the short term. Here we describe the discovery of a conspicuous salmon-pink colouration in the base of bustard feathers and down that has never been reported because of its extraordinarily brief expression. HPLC analyses indicated that its constituent pigments are coproporphyrin III and protoporphyrin IX, which are prone to photodegradation. Accordingly, an experimental exposure of feathers of three bustard species to sunlight produced a rapid disappearance of the salmon-pink colouration, together with a marked decrease in reflectance around 670 nm coinciding with the absorption of porphyrin photoproducts. The disappearance of the salmon-pink colouration can occur in a period as short as 12 min, likely making it the most ephemeral colour phenotype in any extant bird. The presence of this colour trait in males performing sexual displays may thus indicate to females a high probability that the males were performing their first displays and would engage in their first copulations in the breeding season. In dominant males, sperm quality decreases over successive copulations, thus porphyrin-based colouration may evolve as a signal of virginity that allows females to maximize their fitness in lek mating systems.

Animal colouration can serve a number of adaptive functions, such as the protection against mutagenic ultraviolet (UV) radiation and visual communication, including the signalling of genotypic quality to potential mates^1^. Animals colours are generated by the deposition of pigments in integumentary structures such as scales, skin, feathers or hair, and can be synthesized by the animals themselves or acquired from the environment. In some cases, these pigments develop simultaneously with specialized structures (i.e., nanostructures) that interact with light to create distinctive hues^2^. When coloured traits result from the direct action of live pigment cells, for instance the skin, colour expression can change rapidly in response to hormonal and exogenous factors^3^,^4^,^5^. Alternatively, when coloured traits result from the deposition of pigments in inert integumentary structures, such as feathers and hair, colour changes cannot occur until these structures are newly synthesized, modified by physical agents, or until a cosmetic replenishment of pigments occurs^6^,^7^.

Animals thus normally create colours that persist in mid-to-long term time scales. In the relatively exceptional cases when pigment cells produce dynamic colourations, initial colour states are even recovered by activating particular cellular mechanisms, which results in the same colour phenotype being expressed more than once^5^. Such rapid alterations in colour states can be observed in cephalopods and reptiles, and arthropods that produce bioluminescence^8^,^9^. Few studies have considered how the duration of colour expression might influence the evolution of animal colouration. In general, rapidly decaying colour traits are rare in animals. To date, the most rapidly decaying colour traits known are those elicited by hormonal responses derived from breeding events in some amphibians and only last for a few days^10^,^11^. Such changes may be comparable to the dynamic colour changes that occur in flowers, typically in response to pollination^12^. Slight daily colour changes have also been reported in zebrafish, *Danio rerio*, that coincide with mating events^13^. Despite our limited understanding of rapid alterations in colour states, these traits may experience strong sexual selection pressure if they signal to potential mates information about an individual’s breeding status that is only available during a very short period of time^14^,^15^. Expression duration, therefore, may be an overlooked factor influencing the information content and thus the evolution of animal colouration. Here we report a conspicuous colour trait caused by the presence of the pigments porphyrins in the feathers of some birds that irreversibly disappears in minutes after exposure to light, thus representing the most rapidly decaying visual trait in an animal and probably in any living organism. Given the likely role in sexual selection of this trait and its extraordinary short-lived nature, it may be associated with the most valuable signal content sought by signals’ recipients in any biological system so far.

In the 1920’s, the pigments porphyrins were detected in the feathers of birds^16^, and it was later found that their distribution was confined to certain bird groups, most notably owls (Order Strigiformes), nightjars (Order Caprimulgiformes) and bustards (Order Gruiformes)^17^. Porphyrins are very abundant in animal internal tissues and fluids as represented for example by the heme group which colours red blood cells and also in plants as represented by chlorophyll. However, birds are the only animals able to deposit porphyrins in the integument with the only exception of certain fishes and the European hedgehog *Erinaceus europaeus*^18^, aside from anomalous depositions in the human skin during certain diseases (porphyrias). The presence of porphyrins in feathers was not detected because of the visible colouration that they generate, but due to their fluorescent properties (i.e., they emit long-wavelength light when excited by UV light^16^), which are also used to detect their production by acne-related bacteria in the human skin^19^. These fluorescent properties create very conspicuous and intriguing effects^20^, but the perceptible colour that these pigments generate without artificial UV light excitation has been assumed to be negligible^21^. However, we have discovered that porphyrins actually create very conspicuous salmon-pink colouration in non-exposed barbs and barbules of contour feathers and down of bustard species, but it has remained unnoticed until now likely because the colour disappears after a few minutes of exposure to sunlight.

## Results and Discussion

**Figure 1:**
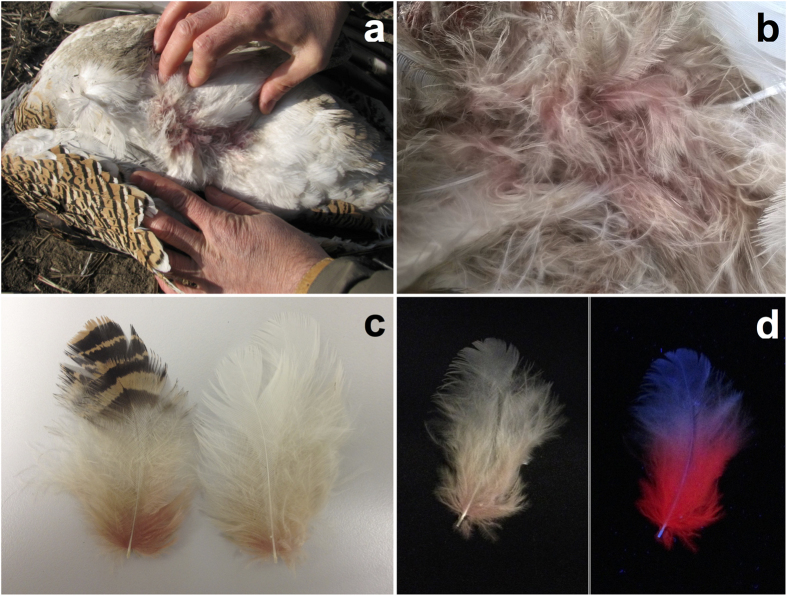
Photographs of great bustard plumage traits with perceptible salmon-pink colouration generated by porphyrins. (**a**) A wild female great bustard found in Bulgaria. (**b**) Details of the belly feathers of a wild male great bustard found in Spain. (**c**) Details of great bustard feathers showing the location of the salmon-pink colouration at the base. (**d**) A great bustard feather with salmon-pink colouration seen under standard daylight (left) and showing intense red fluorescence under UV illumination (right). In this feather, some parts perceived as whitish-greyish under conventional daylight exhibits red fluorescence probably because of a non-complete degradation of porphyrins in those parts.

**Figure 2:**
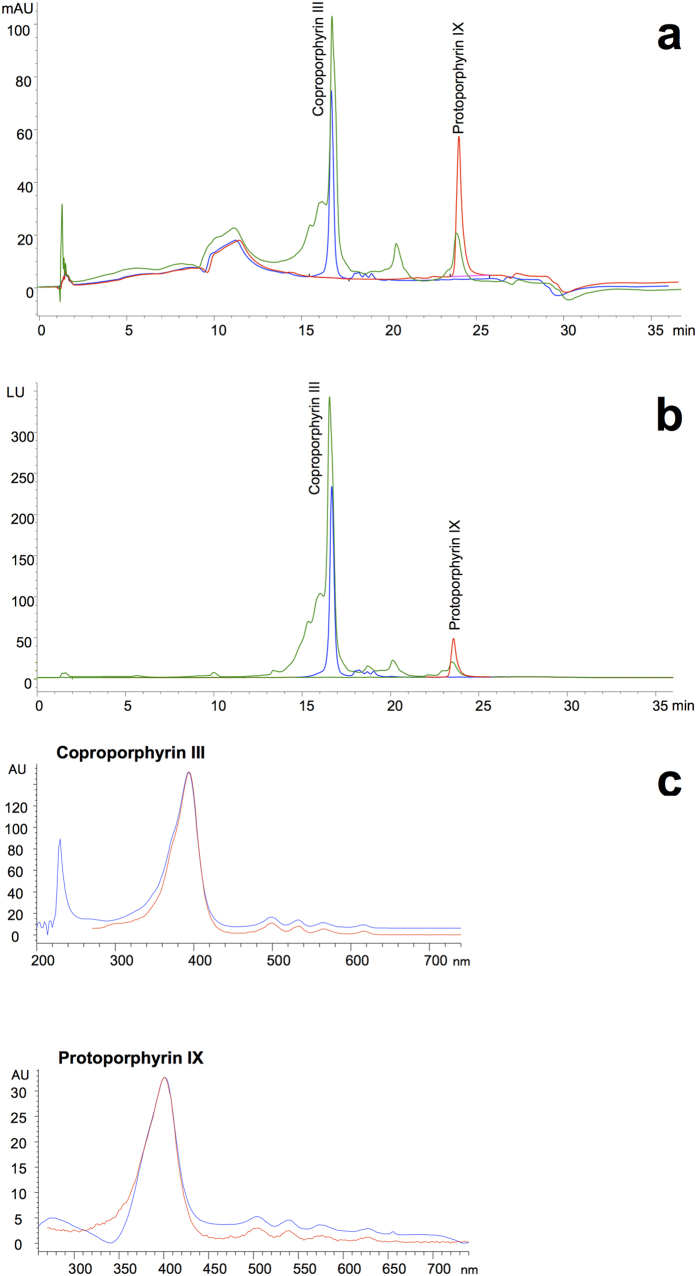
Results of HPLC analyses of porphyrins extracted from great bustard feathers showing salmon-pink colouration in their base. (**a**) Chromatogram of the diode array detector signal from an extraction of two feathers that were orange-black in their distal tips (Fig. 1c left). (**b**) Chromatogram of the fluorescence detector signal from an extraction of two feathers that were white in their distal tips (Fig. 1c right). In (**a** and **b**): blue curves: coproporphyrin III standard; red curves: protoporphyrin IX standard; green curves: extract of great bustard feathers. (**c**) Absorption spectra of standard porphyrins (red curves) and natural porphyrins extracted from the feathers that were orange-black in their distal tips (blue curves).

This novel salmon-pink plumage colouration was first noticed by one of us (R.M.) when a recently shot female great bustard *Otis tarda* was found dead in coastal Dobrudzha, close to Durankulak Lake (Northeast Bulgaria), in January 2012. The specimen exhibited a conspicuous salmon-pink colouration in contour feathers that was only visible when the plumage patch was examined and the base of feathers was exposed (Fig. 1a). We also detected this colouration in the nuptial plumage of an adult male great bustard that was found dead near La Albuera (Southwest Spain) in April 2016 (Fig. 1b). This visible colour trait has never been described in bustards. To identify the pigments underlying this colouration, we conducted chemical analyses of the red feathers of the female great bustard found in Bulgaria. We analysed two belly feathers that were white in their distal tips and two dorsal feathers that were orange-black in their distal tip, all of which showed salmon-pink colouration at the base (Fig. 1c). The salmon-pink portion of these feathers exhibited strong red fluorescence under UV light (Fig. 1d), suggesting that the colour was generated by porphyrins. This was confirmed by high-performance liquid chromatography (HPLC) analyses, which revealed that the salmon-pink portion of feathers contained coproporhyrin III (3,8,13,17-tetramethylporphyrin-2,7,12,18-tetrapropanoic acid) and protoporphyrin IX (3,7,12,17-tetramethyl-8,13-divinylporphyrin-2,18-dipropanoic acid) (Fig. 2), of which the former was more abundant (mean value ± s.e. for the four feathers: 966.50 ± 27.24 pmol/g) than the latter (168.36 ± 4.71 pmol/g).

Porphyrins are photolabile pigments^22^, thus the salmon-pink colouration of great bustard feathers may rapidly degrade under exposure to light and therefore render it difficult to observe. In order to determine the frequency of this colouration pattern among bustards and test hypotheses about colour degradation, we examined captive bustards in a breeding centre of steppe-land birds in Andalusia (Spain). Large numbers of four bustard species are bred and kept in captivity with little direct exposure to sunlight (www.avutardas.com).

**Figure 3:**
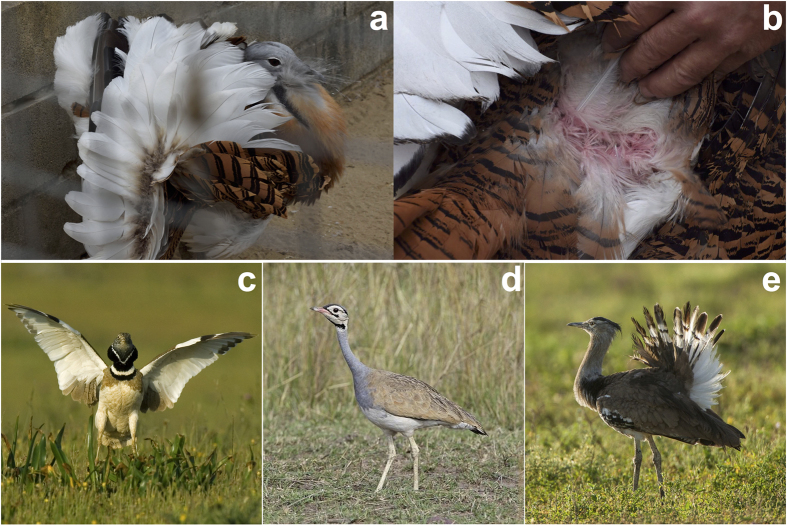
Photographs of the four bustard species examined in captive conditions with little direct exposure to sunlight. (**a**) A male great bustard displaying. (**b**) Detail of the white basal parts of the dorsal feathers of the same male showing salmon-pink colouration generated by porphyrins. (**c**) Male little bustard displaying (photograph by Manuel Calderón Carrasco). (**d**) White-bellied bustard (photograph by Lip Kee; https://flic.kr/p/5iue6Q). (**e**) Kori bustard (photograph by Francesco Veronesi; https://flic.kr/p/pqgEaB). Photographs (**d**) and (**e**) are covered by a CC BY-SA license (https://creativecommons.org/licenses/by-sa/2.0/).

We examined 25 birds of both sexes and ages (first-year birds and adults) for each of the following species of bustards: great bustard, little bustard *Tetrax tetrax*, white-bellied bustard *Eupodotis senegalensis* and kori bustard *Ardeotis kori* (Fig. 3). All these birds had been born in captivity and raised in outdoor aviaries covered by ownings to prevent direct exposure to sunlight. In every individual examined, we observed a conspicuous salmon-pink colouration at the base of the contour body feathers.

**Figure 4:**
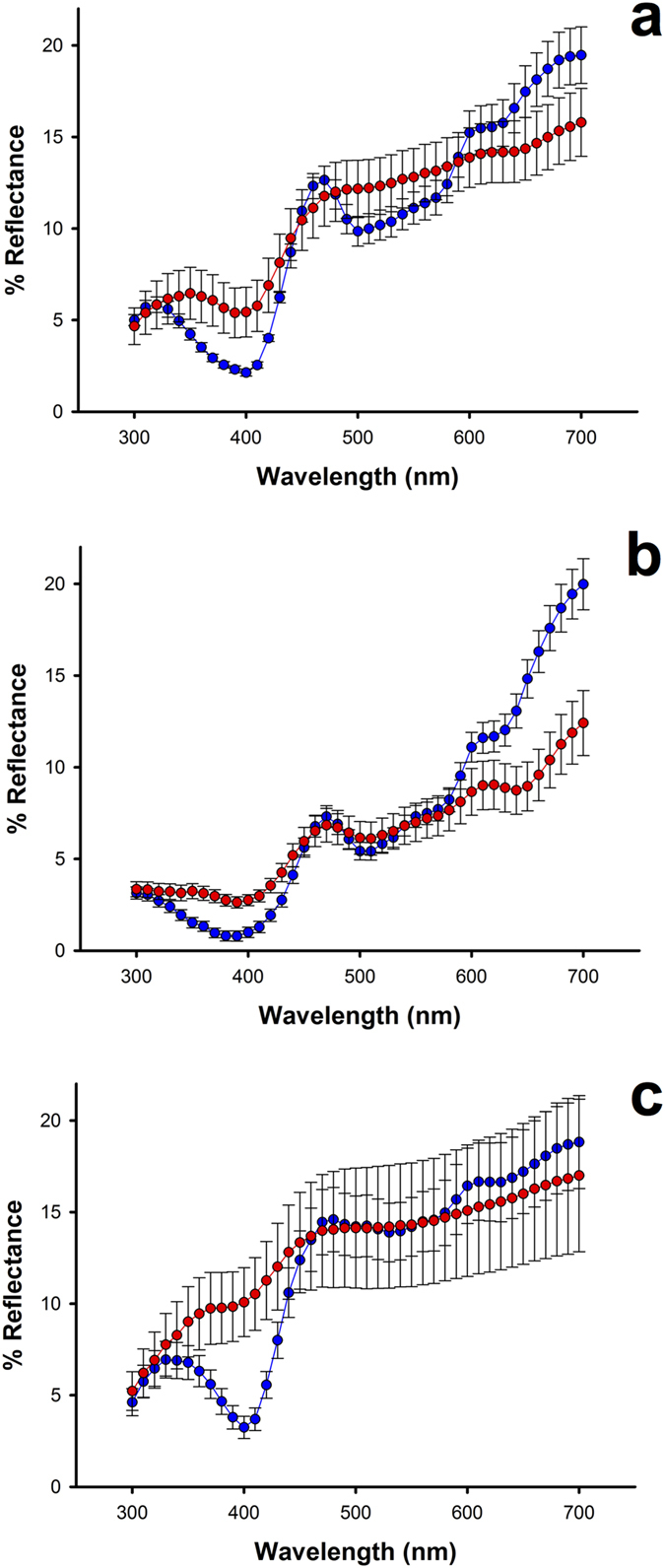
Reflectance spectra of bustard feathers before (blue symbols and lines) and after (red symbols and lines) 3 h experimental exposure to sunlight. Graphs show the mean values of reflectance (±s.e.) corresponding to the porphyrin-based salmon-pink colour patch of seven feathers of great bustard (**a**), nine feathers of white-bellied bustard (**b**) and four feathers of kori bustard (**c**).

To determine whether the salmon-pink colouration degrades after direct exposure to sunlight, we collected body feathers showing the salmon-pink colouration from great bustards, white-bellied bustards and kori bustards, and kept them in dark plastic envelopes. First we obtained the reflectance spectra of the salmon-pink portion of the feathers. We then conducted an experiment to time the colour change that should occur after direct exposure to sunlight. For this, we attached the feathers with adhesive tape to a cardboard, and placed it on the ground in an unshaded location on the rooftop of Doñana Biological Station (Sevilla, Spain). Tests occurred over a 3 h period (from 10 to 13 h) on a cloudless day in April 2016. By the end of the trial, the salmon-pink colour was completely degraded, and a greyish colouration was perceived instead (see electronic supplementary material, movies S1,S2). Indeed, the reflectance measurements taken on the locations of feathers corresponding to the portion previously showing salmon-pink colouration indicated that the main change consisted in a large decrease of reflectance in the red visible spectral range (620–700 nm) and in the disappearance of a reflectance peak in that range, as well as an increase in reflectance around 400 nm (Fig. 4).

Porphyrins are square-planar macrocycles composed of four pyrroles connected by methine bridges to form aromatic rings, a structure that confers them a great capacity to absorb visible light and thus exhibit conspicuous colours, but that also makes them prone to photodestruction when exposed to visible light^23^. This photodegradation most likely occurs as the result of of an opening of the porphyrin ring that leads to an increase of UV light absorbance^24^ and the production of reactive singlet oxygen^25^. The photoproducts that result from this degradation process have a characteristic absorption band around 670 nm, as previously reported for protoporphyrin IX^22^. Interestingly, this band also occurs in the spectral region where we observed the largest decrease in feather reflectance after exposure to sunlight (Fig. 4). Thus, the salmon-pink colouration of bustard feathers and down rapidly disappears after direct exposure to sunlight because of the photobleaching of porphyrins. Photobleaching studies of protoporphyrin IX have shown that this photooxidative process can occur within 30 min of exposure to light, as revealed by a decrease of fluorescence intensity^26^. We therefore hypothesized that the disappearance of the salmon-pink colour of bustard feathers may occur even more rapidly than the 3 h-period tested previously. To address this, we video recorded bustard feathers showing salmon-pink colouration from the instant they were directly exposed to sunlight and found that the salmon-pink colour was no longer perceptible after 25 min of sunlight exposure in white-bellied bustard feathers and after 12 min in great bustard feathers (electronic supplementary material, movies S1,S2).

With an effective expression of only a few minutes and an irreversible nature (the plumage cannot recover its basal salmon-pink colour until new feathers are developed during moult), the porphyrin-based salmon-pink colouration of bustard feathers is, to our knowledge, the most ephemeral colour phenotype in birds and probably in any living organism described so far. This decay rate may only be comparable to olfactory signals generated by volatile molecules^27^. Porhyrins are not thought to confer perceptible colour to the animal integument, thus research has primarily focused on testing adaptive hypotheses for the fluorescence patterns exhibited by these pigments^18^. However, under normal daylight conditions, the contribution of fluorescence to radiance is negligible in other pigments present in the feathers of parrots^28^, which is likely to be the case for feather porphyrins as well. Our findings indicate that the biological function of porphyrins in the animal integument, if any, might not be fulfilled by their fluorescence properties but instead by their visible properties which create a conspicuous salmon-pink colouration that had been unnoticed because of its extraordinary short life derived from its photolability.

This study reveals the existence of a novel conspicuous animal colour trait with unprecedented properties that should stimulate research to elucidate possible adaptive functions. We believe the latter is highly probable, as numerous vertebrates including humans show a sensory bias towards red which makes that traits so coloured are often associated with dominance and sexual attractiveness^29^. Porphyrins are intimately linked to the synthesis of red blood cells, as haemoglobin is formed after the addition of an iron ion to protoporhyrin IX followed by protein bonding^30^. Porphyrins thus confer oxygenated blood its characteristic red colour, which turns to blue when the oxygen is lost. Interestingly, blood oxygenation level is reflected by the redness of skin, which increases with perception of health and attractiveness in humans^31^. The salmon-pink colour of bustard feathers, which is produced by porphyrins and thus associated with blood synthesis, may transfer similar information to conspecifics about health status.

**Figure 5:**
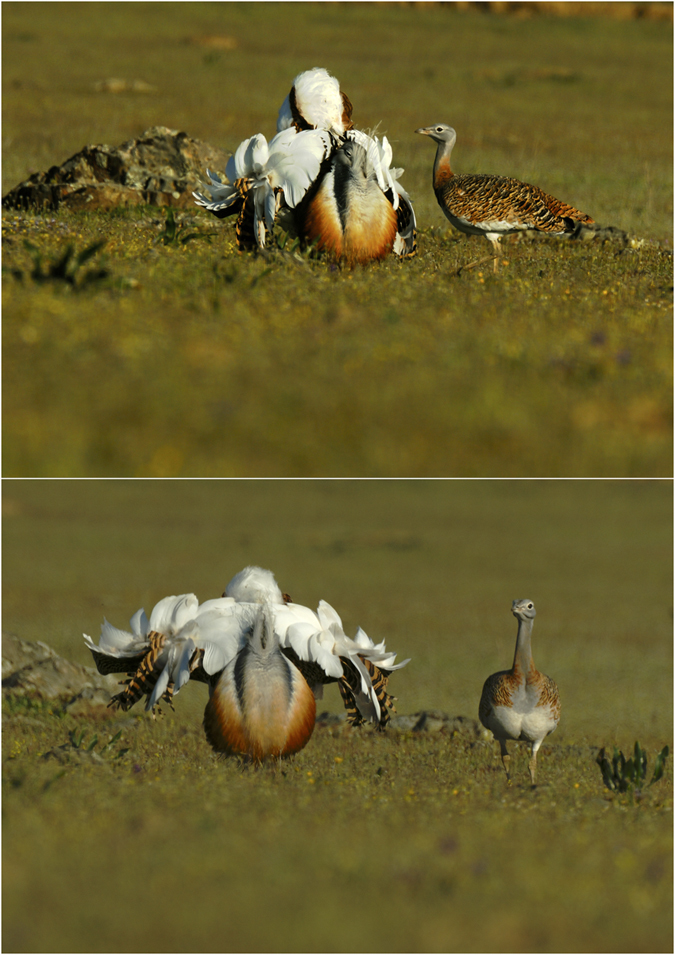
Photographs of a male great bustard (left) performing sexual display while the plumage traits are closely assessed by a female (right) in a wild population in La Serena (Extremadura, Spain). Photographs by Manuel Calderón Carrasco.

Knowledge on the mating behavior of the great bustard reinforces the interesting possibility that this trait functions as a visual signal. Mate selection in great bustards occurs through a lek strategy, consisting in the grouping of males to display complex visual exhibitions of plumage that females assess to select their mates. In this system, approximately 10% of males achieve successful copulation attempts^32^. Although the salmon-pink colour of great bustard plumage is only visible in the base of the feathers (Figs 1 and 3b and electronic supplementary material, movies S1,S2), the sexual exhibition of great bustard males is extraordinarily exaggerated and entirely visual^33^. Specifically, in this display males inflate the oesophagus and adopt a posture in which their feathers are turned over in a manner that reveals their white underparts and base (Figs 3a and 5). Females assess the plumage characteristics of males^32^ from a very short distance (Fig. 5), a situation in which they can potentially observe the salmon-pink colour of male feathers (Fig. 3b). As this colour trait rapidly decays under sunlight, males can only use it during a few display bouts, thus functioning similar to a sand clock. Mating in great bustards occurs in April and feathers are moulted between July and October^34^, thus salmon-pink feathers cannot be replaced in a single mating period. Interestingly, great bustard males direct their turned feathers towards the sun during displays^35^, which might increase the degradation speed of the red colouration. Other bustard species, including the three remaining species in which we have discovered porphyrin-based salmon-pink colouration, exhibit similar visual sexual displays with varying levels of complexity. These displays include strategies to increase visibility and attract females from a distance and can include acoustic signals^36^, but females always evaluate males from a close distance which coincides with the highest levels of display complexity^37^.

Thus, any female that finds salmon-pink colouration in a displaying male may determine that the male has not displayed for a long period of time and therefore may not have copulated previously in the current mating season, i.e. it may be a signal of virginity. This information would be essential in order for females to maximize their fitness in lek mating systems, where females do not obtain any resource benefits from copulating with males apart from gametes^38^. It has been demonstrated in dominant male fowl that sperm quality, which determines fertilization success, decreases over successive copulations^39^. Therefore, females that observe salmon-pink colouration in dominant males (which attain the greatest number of successful copulations^32^), might be the first to copulate in the breeding season and therefore obtain higher quality sperm. The presence of porphyrin-based feather colouration in female bustards may arise as a genetically correlated result of selection on males^40^. To test this hypothesis, further experimental studies need to be conducted. Specifically, the salmon-pink colouration could be experimentally removed from the plumage of some captive male bustards directly exposed to sunlight, while other males could be provided with artificial coverage to maintain the expression of salmon-pink colour. These males could then be introduced to single females to determine whether females prefer males with preserved salmon-pink colouration. Additionally, it should be determined if the fertilization success of eggs laid by female bustards decreases with the number of copulations achieved by the males. Studies in wild populations of bustards will also be useful to determine whether males exhibit salmon-pink colouration during the first sexual displays in the breeding season.

A signal of virginity is not expected to evolve as a signal of genotypic quality, because the duration of its expression depends upon an environmental factor. Instead, a signal of virginity is likely to evolve as an amplifier (of virginity), i.e. a honest signal whose honesty is not given by costs but by their design because they improve the perception of other signals or cues^41^,^42^. The reproductive state of male birds (virgin/no virgin) would be a cue, i.e. a trait that, by contrast to signals, confers no costs on the fitness of signalers and may not be heritable or evolve by natural selection alone^41^. In this case, the salmon-pink colouration generated by porphyrins may be an amplifier of a cue (virginity). Virgin male bustards would thus benefit (in terms of copulation success) from performing the sexual display that reveals the salmon-pink colouration and amplifies their virgin state, while non-virgin males may achieve a lower copulation success for showing their lack of salmon-pink colouration and amplifying their non-virgin state. However, non-virgin males would still achieve greater reproductive success by signaling their non-virgin as opposed to not signaling at all^41^. The fact that amplifiers of cues must be conspicuous and stereotyped to evolve^43^ supports the prediction that the salmon-pink colouration is an amplifier of virginity, as it is a conspicuous colouration that is exhibited by males during stereotyped displays. It must be noted that the salmon-pink colouration is not the only trait in male bustards that females use to determine their mating decisions and it may interact with other signals and cues, such as body size^32^,^33^. Future studies should investigate the relative importance of these different signals as well as their interactions for shaping female mate choice.

The synthesis by living organisms of all pigments that confer colour in nature can be grouped into just three common metabolic routes^44^: the levulinic, mevalonic and shiskimic routes. In birds, the majority of the brightest colours produced are due to carotenoids, which are generated in the mevalonic route. This route is also responsible for the most prevalent chemical attractors, such as hormones and pheromones. The levulinic route is the one leading to the synthesis of porphyrins, and although it has been extensively investigated in plants due to the essential function of chlorophyll in these organisms, it has been overlooked as a contributing factor to the appearance of animals. Our findings show that the levulinic route also leads to conspicuous and ephemeral integument colourations, opening a new avenue for the study of visual communication in which the expression duration of colour may have a key evolutionary role.

## Material and Methods

All methods were carried out in accordance with relevant guidelines and regulations in Spain.

### Analysis of porphyrins in feathers

Great bustard feathers were analysed by HPLC following a protocol modified from Mateo *et al*.^45^, and with fluorescence detection. Feather barbs corresponding to the salmon-pink colour patch were excised from the rachis, trimmed and treated with HCl 3N and acetonitrile. The mixture was shaken, incubated at dark for 30 min and then sonicated at cold for 15 min. Extractions were centrifuged at 10,000 g and 4 °C for 5 min, filtered and added to HPLC vials. A HP1200 series quaternary pump, autosampler, column oven and diode array detector were used (Seeltze, Germany). All the chromatographic conditions and quantification were controlled using ChemStation software (ver. B.04.02). A Waters (Milford, MA, USA) Spherisorb ODS 2 (5 μm particle size, 4.6 mm × 100 mm) chromatographic column was used. The flow rate was 0.8 ml/min and a solvent gradient elution was used. The initial mobile phase composition was methanol 25% and ammonium acetate (10 mM, pH 5.16) 75% for 4 min. The solvent gradient consisted in a 20 min linear change to 100% methanol, followed by 2 min at these conditions. At this moment the phase composition returned to the initial conditions in 5 min and remained at this status for another 5 min. The total run time was 36 min. The column was maintained at 60 °C and detection by a fluorescence detector was made with excitation wavelength of 403 nm and emission wavelength of 603 nm, also including detection by a diode array detector at 402 nm, as this coincides with the maximum absorbance of both standard and extracted porphyrins (Fig. 2c). Standard porphyrins were purchased from Frontier Scientific Ltd. (Carnforth, UK).

### Analysis of feather colouration

The salmon-pink colour expression of bustard feathers was analysed by reflectance spectrophotometry before and after experimental exposure to sunlight. We used an Ocean Optics (Dunedin, FL) Jaz spectrophotometer (range 220–1000 nm) with ultraviolet (deuterium) and visible (tungsten-halogen) lamps and a bifurcated 400 μm fiber optic probe. The fiber optic probe both provided illumination and obtained light reflected from the sample, with a reading area of ca. 1 mm^2^. Feathers were mounted on a light absorbing foil sheet (Metal Velvet coating, Edmund Optics, Barrington, NJ) to avoid any background reflectance. Measurements were taken at a 90° angle to the sample. All measurements were relative to a diffuse reflectance standard tablet (WS-1, Ocean Optics), and reference measurements were frequently made. An average spectrum of six readings on different points of the red colour patch was obtained for each feather, removing the probe after each measurement. The same was made after the exposure of feathers to sunlight, taking the measurements at the same points of feathers. The analyses were made on individual feathers separately, and mean spectra were then calculated. Reflectance curves were determined by calculating the median of the percent reflectance in 10 nm intervals.

## Additional Information

**How to cite this article:** Galván, I. *et al*. Porphyrins produce uniquely ephemeral animal colouration: a possible signal of virginity. *Sci. Rep.* **6**, 39210; doi: 10.1038/srep39210 (2016).

**Publisher's note:** Springer Nature remains neutral with regard to jurisdictional claims in published maps and institutional affiliations.

## Supplementary Material

Supplementary Information

Supplementary Movie S1

Supplementary Movie S2
