# Supplementary material for: Porphyrins produce uniquely ephemeral animal colouration: a possible signal of virginity
Source: Sci Rep. 2016 Dec 15;6:39210. doi: 10.1038/srep39210 (PMC5156940; doi:10.1038/srep39210)
Supplement: Supplementary Information [file srep39210-s1.pdf]

Porphyrins produce uniquely ephemeral animal colouration: a possible signal of virginity

Ismael Galván, Pablo R. Camarero, Rafael Mateo and Juan J. Negro

Legends for electronic supplementary material:

Movie S1. Time-lapse video of a white-bellied bustard dorsal feather showing the disappearance of salmon-pink colouration in 25 min of exposure to sunlight due to photodegradation of porphyrins.

Movie S2. Time-lapse video of three great bustard belly feathers showing the disappearance of salmon-pink colouration in 12 min of exposure to sunlight due to photodegradation of porphyrins.
